# Biophysical Characterization of the Oligomeric States of Recombinant Immunoglobulins Type-M and Their C1q-Binding Kinetics by Biolayer Interferometry

**DOI:** 10.3389/fbioe.2022.816275

**Published:** 2022-05-24

**Authors:** Anne Chouquet, Andrea J. Pinto, Julia Hennicke, Wai Li Ling, Isabelle Bally, Linda Schwaigerlehner, Nicole M. Thielens, Renate Kunert, Jean-Baptiste Reiser

**Affiliations:** ^1^ Institut de Biologie Structurale, UMR 5075, Univ. Grenoble Alpes, CEA, CNRS, IBS, Grenoble, France; ^2^ Department of Biotechnology, University of Natural Resources and Life Sciences, Vienna, Austria

**Keywords:** immunoglobulins, IgM, complement, C1q, recombinant expression, biophysical characterization, protein–protein interaction, biolayer interferometry

## Abstract

Immunoglobulins type-M (IgMs) are one of the first antibody classes mobilized during immune responses against pathogens and tumor cells. Binding to specific target antigens enables the interaction with the C1 complex which strongly activates the classical complement pathway. This biological function is the basis for the huge therapeutic potential of IgMs. But, due to their high oligomeric complexity, *in vitro* production, biochemical characterization, and biophysical characterization are challenging. In this study, we present recombinant production of two IgM models (IgM617 and IgM012) in pentameric and hexameric states and the evaluation of their polymer distribution using different biophysical methods (analytical ultracentrifugation, size exclusion chromatography coupled to multi-angle laser light scattering, mass photometry, and transmission electron microscopy). Each IgM construct is defined by a specific expression and purification pattern with different sample quality. Nevertheless, both purified IgMs were able to activate complement in a C1q-dependent manner. More importantly, BioLayer Interferometry (BLI) was used for characterizing the kinetics of C1q binding to recombinant IgMs. We show that recombinant IgMs possess similar C1q-binding properties as IgMs purified from human plasma.

## 1 Introduction

Immunoglobulins of type M (IgMs) are the first Igs produced and secreted by B cells during early infection and immune reaction stages in adults. They are highly oligomeric polypeptides composed of a heavy chain (H) and a light chain (L). Each chain contains an N-terminal variable domain and one (for the L chain) or four (for the H chain) C-terminal constant domains. In serum, IgMs are mainly found as an assembly of H_2_L_2_ protomer subunits in a pentameric form (H_2_L_2_)_5_ containing an additional covalently linked peptide called the joining (J) chain, but also to a lesser extent (less than 5%), in hexameric form (H_2_L_2_)_6_, devoid of J chain (for reviews Gong and Ruprecht, 2020; Jones et al., 2020; Keyt et al., 2020). IgMs are also highly glycosylated with 5–6 different N-linked glycosylation chains that can account for up to 10–15% of their total molecular weight (Arnold et al., 2005). The overall assembly of IgMs is now revealed by modern high-resolution electron microscopy (EM) methods. They have been observed as star-shaped structures with a compact Fc core and orbiting, labile, and flexible Fab arms. While hexamers have the expected 6-fold symmetry, the pentamers possess a pseudo-hexameric symmetry in which five IgM protomers occupy five of the six symmetric positions and the J chain the sixth position (Hiramoto et al., 2018; Li et al., 2020; Kumar et al., 2021).

In addition to their antigen recognition function, IgMs fulfill their immune effector function by being one of the most potent activators of the classical pathway of the complement system. The binding of complexes between specific surface-exposed antigens and IgMs to the first recognition component of the classical pathway (CP), the C1q molecule, initiates a regulated amplifying proteolytic cascade, which in turn enables the formation of the membrane attack complex and the elimination of the pathogen or infected cell targets (Ricklin et al., 2010; Cedzyński et al., 2019). C1q is found in high concentration in human serum as part of the CP initiation C1 complex together with the C1r_2_C1s_2_ pro-enzyme tetramer. It is a 460-kDa highly flexible glycoprotein assembled from 18 polypeptide chains of 3 types (A, B, and C). These are organized into six ABC heterotrimers forming six Ig recognition domains, the globular heads (gC1q), attached to collagen-like regions (CLR). The C1q assembly has been depicted as a “bouquet of tulips” or an “Eiffel tower” (Kishore and Reid, 2000; Diebolder et al., 2014; Sharp et al., 2019).

Methods to measure direct binding characteristics of Igs to C1q as functional quality attributes are rather limited in number (reviewed in Harboe et al., 2011). Traditional methods employ enzyme-linked immunosorbent assay (ELISA) to measure equilibrium and end-point deposition of Igs on coated C1q and *vice versa* (exemplified in Duncan and Winter, 1988; Idusogie et al., 2000). More recently, label-free and real-time methods such as surface plasmon resonance (SPR) or biolayer interferometry (BLI) have been used to characterize the binding kinetics between C1q and IgGs (Moore et al., 2010; Patel et al., 2015; Zhou et al., 2018) or IgMs (Bally et al., 2019).

In the present study, we report productions of our IgM models, IgM617 and IgM012, in pentameric and hexameric forms and their biochemical and functional quality characterization using analytical ultracentrifugation (AUC), size exclusion chromatography coupled to multi-angle laser light scattering (SEC-MALLS), mass photometry (MP), transmission electron microscopy (TEM), and in-house ELISA for detection of complement activation. More importantly, we also report protocols to evaluate their bindings to C1q with BLI and compare their binding kinetics.

## 2 Materials and Methods

### 2.1 Immunoglobulins Type-M Genetic Constructs

IgM617 is originally expressed by the EBV-transformed B cell line HB617 and directed against glycosphingolipids overexpressed on the tumor cell surfaces to elicit cytotoxic T-cells (Vorauer-Uhl et al., 2010). IgM012 was developed after a class switch of an HIV broadly neutralizing human IgG-targeting mannose carbohydrate structures of the HIV1 envelope protein gp120 (Wolbank et al., 2003). Light chain (L), heavy chain (H), and joining chain (J) cDNAs of both IgM models were codon-optimized and sub-cloned into separate pIRES vectors as described in Chromikova et al. (2015b), into pCEP4 vectors as described in Hennicke et al. (2017), and into pcDNA3.1(+) vectors. For the last constructs, IgM chain cDNAs were amplified by the polymerase chain reaction (PCR) using conventional protocols and the pIRES constructs as templates. Additional flanked NheI and BamHI restriction sites were also introduced by PCR. The genes were then inserted individually with classical ligation techniques into pcDNA3.1(+) vectors (Invitrogen) containing a resistance cassette for either Geneticin (H chain), Zeocin (L chain), or hygromycin (J chain).

### 2.2 Cell Lines and Expression of Immunoglobulins Type-M Models

IgM617-HLJ and IgM012-HLJ were expressed using 1) CHO DG44 cell lines by co-transfection with pIRES constructs and generation of stable cells lines (Chromikova et al., 2015b), 2) HEK293E cell lines after co-transfection with pCEP4 vectors and transient expression (Hennicke et al., 2019), or 3) HEK293F after co-transfection or serial transfections with pcDNA3.1(+) vectors and generation of stable cell lines.

Transfection and cultivation conditions of CHO DG44 and HEK293E cell lines have been described previously (Chromikova et al., 2015b; Hennicke et al., 2020). To generate stable HEK293F cell lines expressing either IgM617-HL or IgM012-HL, cells were simultaneously co-transfected with H-chain- and L-chain-containing pcDNA3.1(+) plasmids using 293 fectin according to the manufacturer’s protocol (Invitrogen) and grown in FreeStyle 293 expression medium supplemented with 400 μg/ml G418 (Invitrogen) and 10 μg/ml Zeocin (Invitrogen). These cells were then transfected with J-chain-containing pcDNA3.1(+) plasmids in the same way, and the stable transfectants producing either IgM617-HLJ or IgM012-HLJ were generated using cultivation in a medium supplemented with additional 100 μg/ml hygromycin (Sigma-Aldrich). The stable cells expressing each IgM construct were then cultivated in Freestyle 293 expression medium under antibiotic pressures and passaged every 3–4 days when cell density approached 3.10^6^ cells/ml.

### 2.3 Recombinant Immunoglobulins Type-M Purification

All IgMs were purified from harvested supernatants according to Hennicke et al. (2017). In brief, POROS CaptureSelect^TM^ IgM Affinity Matrix (Thermo Fisher Scientific) was used for affinity chromatography. Culture supernatants were directly applied to the packed column, and the IgMs were eluted with 1 M arginine and 2 M MgCl_2_, pH 3.5 or pH 4.0. The collected fractions were immediately neutralized with 1 M Tris pH 8.5. The IgM-containing fractions were then pooled, dialyzed against the next-step equilibration buffer and concentrated. For a second purification step consisting of a size exclusion chromatography (SEC), they were applied on a Superose^TM^ 6 increase 10/300 or 16/600 column (GE Healthcare/Cytiva) equilibrated in 0.1 M sodium phosphate pH 7.4, 0.2 M NaCl or in 0.025 M Tris–base, 0.137 M NaCl, and 0.003 M KCl, pH 7.4 at a flow rate of 0.5 ml/min. Highly multimeric IgMs were eluted as a single peak separated from lower or higher oligomeric states and from nucleic acid contaminants. Identification of purified IgMs by SDS-PAGE was performed as described in Vorauer-Uhl et al. (2010) using native PAGE 3–12% Bis–Tris gels followed by Coomassie Blue staining.

### 2.4 C1q Purification From Plasma

C1q was purified from human serum according to the well-established protocol. Briefly, IgG-ovalbumin-insoluble immune aggregates were prepared as described by Arlaud et al. (1979). Human serum, obtained from the Etablissement Français du Sang Rhône-Alpes, was incubated after clarification by centrifugation with immune aggregates on ice for 45 min. C1/immune complexes were then collected by centrifugation and extensively washed with 20 mM Tris, 120 mM NaCl, and 5 mM CaCl_2_ at pH 7.4. Following C1r and C1s release by washing with buffer containing EDTA, C1q was eluted from immune aggregated with 50 mM Tris and 700 mM NaCl at pH 10.0 and separated from the immune complex by centrifugation. C1q samples were further purified to homogeneity by CM-cellulose chromatography.

### 2.5 Analytical Ultracentrifugation

Sedimentation velocity Analytical ultracentrifugation (sv-AUC) experiments were conducted in an XLI analytical ultracentrifuge (Beckman, Palo Alto, CA) using an ANTi-60 rotor, double channel Ti center pieces (Nanolytics, Germany) of 12- or 3-mm optical path length equipped with sapphire windows and the reference channel being typically filled with the sample solvent. Acquisitions were performed overnight at 4°C and at 20,000 rpm (32,000 g) using absorbance (280 nm) and interference detection. Data processing and analysis were completed using the program SEDFIT (Schuck, 2000) from P. Schuck (NIH, United States), REDATE (Zhao et al., 2015) and GUSSI (Brautigam, 2015) from C. Brautigam (United States), and using standard equations and protocols described previously (Salvay et al., 2008; Le Roy et al., 2013, 2015).

### 2.6 Size Exclusion Chromatography—Multi-Angle Laser Light Scattering Analyses

SEC combined with online detection by MALLS, refractometry, and UV-Vis was used to measure the absolute molecular mass in solution. The SEC runs were performed using a Superose^TM^ 6 increase 10/300 column (GE Healthcare/Cytiva) equilibrated in 0.025 M Tris–Base, 0.137 M NaCl, and 0.003 M KCl, pH 7.4. Protein sample of 50 μl, concentrated to about 1 mg/ml, was injected with a constant flow rate of 0.5 ml/min, and separation was performed at room temperature. Online MALLS and differential refractive index detection were performed using a DAWN-HELEOS II detector (Wyatt Technology Corp.) with a laser emitting at 690 nm and an Optilab T-rEX detector (Wyatt Technology Corp.), respectively. Weight-averaged molar mass determination was performed with ASTRA6, using the “protein conjugate” module. The following refractive index increments and UV-Vis absorbance values were used: *dn/dc* protein = 0.185 ml/g; *dn/dc* glycosylation = 0.15 ml/g; A_280_ = 1.38 ml/mg.cm.

### 2.7 Mass Photometry

Coverslips (high-precision glass coverslips, 24 × 50 mm^2^, No. 1.5H; Marienfeld, Lauda-Königshofen, Germany) were cleaned by sequential sonication in Milli-Q H_2_O, 50% isopropanol (HPLC grade)/Milli-Q H_2_O, and Milli-Q H_2_O (5 min each), followed by drying with a clean nitrogen stream. To keep the sample droplet in shape, reusable self-adhesive silicone culture wells (Grace Bio-Labs reusable CultureWell^TM^ gaskets) were cut into 4–10 segments. To ensure proper adhesion to the coverslips, the gaskets were dried well using a clean nitrogen stream. To prepare a sample carrier, gaskets were placed in the center of the cleaned coverslip and fixed tightly by applying light pressure with the back of a pipette tip. Protein landing was recorded using a Refeyn One^MP^ (Refeyn Ltd., Oxford, United Kingdom) MP system by forming a droplet of each IgM sample at a final concentration of 10 nM in 0.025 M Tris–Base, 0.137 M NaCl, 0.003 M KCl, pH 7.4. Movies were acquired for 120 s (12,000 frames) with Acquire^MP^ (Refeyn Ltd., v2.1.1) software using standard settings. Contrast-to-mass (C2M) calibration was performed using a mix of proteins with molecular weights of 66, 146, 500, and 1,046 kDa. Data were analyzed using Discover^MP^ (Refeyn Ltd., v2.1.1), and analysis parameters were set to T1 = 1.2 for threshold 1. The values for number of binned frames (nf = 8), threshold 2 (T2 = 0.25), and median filter kernel (=15) remained constant. The mean peak contrasts were determined in the software using Gaussian fitting. The mean contrast values were then plotted and fitted to a line. The experimental masses were finally obtained by averaging replicates using independent recombinant IgM preparations (2–4), and errors were the standard deviation.

### 2.8 Transmission Electron Microscopy

About 4 μl of diluted IgM samples (60–80 ng) were applied to a carbon film evaporated onto a mica sheet. The carbon film was then floated off the mica in ∼100 µl 2% sodium silicotungstate (SST, Agar Scientific) and transferred onto a 400 mesh Cu TEM grid (Delta Microscopies). Images were acquired using a CETA camera on a Tecnai F20 TEM microscope operating at 200 keV.

### 2.9 Complement Activation—Enzyme-Linked Immunosorbent Assays

The activation of the classical complement pathway was monitored by an ELISA based on the detection of C4b deposition according to Bally et al. (2019); Hennicke et al. (2020). In brief, 200 ng of IgMs diluted in PBS were adsorbed on a MaxiSorp 96-well plate (Thermo Fisher Scientific) by incubating overnight at 4°C. After washing, unspecific binding was prevented by saturation with PBS complemented with 2% bovine serum albumin (BSA, Sigma-Aldrich) for 1 h at 37°C. Replicate wells were then incubated with either normal human serum (NHS) diluted 25 times in a buffer containing 5 mM Veronal, 150 mM NaCl, 5 mM CaCl_2_, 1.5 mM MgCl_2_ at pH 7.4, C1q-depleted serum (NHSΔ, CompTech) diluted 25 times, or NHSΔ diluted 25 times and reconstituted with purified human C1q (4 μg/ml) for 1 h at 37°C. NHS was obtained from the Etablissement Français du Sang Rhône-Alpes (agreement number 14-1940 regarding its use in research). The reaction was stopped by washing with a buffer containing 5 mM Veronal, 150 mM NaCl, and 5 mM EDTA at pH 7.4. Deposited cleaved C4 form was detected with a rabbit anti-human C4 polyclonal antibody (Siemens), an anti-rabbit-HRP antibody conjugate (Sigma-Aldrich), addition of TMB (Sigma-Aldrich), and a CLARIOstar plate reader (BMG Labtech). Polyclonal IgM isolated from human serum (Sigma-Aldrich) was used as a control. Blank wells were prepared and processed similarly to wells coated with IgMs but incubated with buffer instead of NHS samples. Reported values were obtained by normalizing each data set (polyclonal IgM/NHS defined as 100) after blank subtraction and by averaging data obtained in replicated assays using independent recombinant IgM preparations (between 2 and 4); reported errors were the standard deviation of the replicates.

### 2.10 BioLayer Interferometry

BLI experiments were performed on an OctetRED96e from Pall/FortéBio and were recorded with the manufacturer’s software (Data Acquisition v11.1). All protein samples were buffer exchanged against either 0.01 M Na_2_HPO_4_, 0.0018 M KH_2_PO_4_, 0.137 M NaCl, and 0.0027 M KCl at pH 7.4 (phosphate-buffered saline, PBS) or 0.025 M Tris, 0.15 M NaCl, and 0.003 M KCL at pH 7.4 (Tris-buffered saline, TBS) with Zeba Spin Desalting columns (Thermo Fisher Scientific) prior to loading. Commercial AR2G (amine coupling), SA (streptavidin), APS (aminopropylsilane), Protein A, and Protein L biosensors (Pall/FortéBio) or lab-made IgM-specific biosensors were tested. For the latter, mouse or goat anti-μ chain antibodies (Invitrogen) or CaptureSelect anti-IgM nanobody (Thermo Fisher Scientific) were biotinylated using NHS-PEG4-biotin EZ-link kit (Thermo Fisher Scientific) and captured onto SA biosensors. For their capture on AR2G biosensors, IgM samples were diluted in 0.01 M sodium acetate at pH 4, 4.5, 5, 5.5, or 6 (best capture at pH 6) and C1q in either 0.01 M sodium acetate at pH 4, 4.5, 5, or 5.5, 0.01 M MES at pH 6 or 6.5, or 0.01 M HEPES at pH 7 or 7.5 (best capture at pH 7.5). For their capture on SA biosensor, IgM or C1q samples were biotinylated using NHS-PEG4-biotin EZ-link kit (Thermo Fisher Scientific) according to manufacturer’s conditions. No chemical treatments were applied before capturing on APS, Protein A, Protein L, or IgM-specific biosensors. AR2G biosensors were activated by dipping them in a mix of 10 mM N-hydroxysulfosuccinimide (s-NHS) and 20 mM 1-Ethyl-3-3dimethylaminopropyl (EDC) for 300 s prior to capture and quenched with 1 M ethanolamine pH 8.5 for 300 s after capture. In the case of APS biosensors, they were quenched with 50 μg/ml BSA solution for 600 s. Analyses were performed in 0.2 ml per well in black 96-well plates (Nunc F96 MicroWell, Thermo Fisher Scientific) at 25°C at 1,000 rpm agitation. Biosensors were pre-wetted in 0.2 ml PBS, 0.02% Tween-20, or analysis buffer for 10 min, followed by equilibration in pre-wetting buffer for 120 s. All ligand samples were applied at concentrations between 10 and 50 μg/ml and loaded according to the capture chemistry for between 300 and 600 s, followed by an additional equilibration step of 120 s or more in analysis buffer. For association and dissociation, all analyte samples were diluted at concentrations between 10 and 100 nM in TBS complemented with 0.002 M CaCl_2_ and 0.02% Tween-20 as analysis buffer. Association phases were monitored during dipping the functionalized biosensors in analyte solutions for 180–300 s, and the dissociation phases were monitored in the analysis buffer for 180–300 s. To assess and monitor unspecific binding of analytes, measurements were performed using biosensors treated with the same protocols but replacing ligand solutions with analysis buffer complemented or not with 1% BSA. Since C1q unspecific binding levels were similar, further analyses were performed without BSA. Kinetics analyses were performed using Protein L biosensors which were functionalized with each IgM sample diluted at 30 μg/ml for 600 s until reaching a spectrum shift between 5.5 and 7.0 nm. The association phase of plasma C1q was monitored for 300 s with the diluted sample in analysis buffer at concentrations between 0 and 100 nM and dissociation phase for 600 s. All measurements were performed in replicates (between 2 and 4) using independent recombinant IgM preparations and loadings. Kinetics data were processed with the manufacturer’s software (Data analysis HT v11.1). Signals from the reference biosensor and zero-concentration sample were subtracted from the signals obtained for each functionalized biosensor and each analyte concentration. Resulting specific kinetics signals were then fitted using a global fit method and a 2:1 heterogeneous ligand model. Reported kinetics parameter values were obtained by averaging the values obtained with replicated assays and reported errors as the standard deviation.

## 3 Results

### 3.1 Recombinant Expression by HEK293F and Purification of Different Oligomeric Forms of Immunoglobulins Type-M

Previously, the production of recombinant IgM models using different stable and transient expression systems in mammalian cells has been intensively studied (Wolbank et al., 2003; Vorauer-Uhl et al., 2010; Chromikova et al., 2015a; Chromikova et al., 2015b; Hennicke et al., 2017; Hennicke et al., 2019; Hennicke et al., 2020). Here, we present an additional production system for the two IgM constructs, IgM617 and IgM012, using stabilized HEK293F cell lines. cDNAs of H, L, and J chains from both models were individually subcloned in pcDNA3.1(+) (Supplementary Figure S1). HEK293F cells were transfected with the three H, L, and J pcDNA3.1(+) constructs to obtain IgM samples in pentameric forms (IgM617-HLJ and IgM012-HLJ) or with only H and L vector constructs to obtain IgM samples in hexameric forms (IgM617-HL and IgM012-HL). After selection and culture expansion, recombinant IgMs were purified from culture media using the optimized protocol established by Hennicke et al. (2017). As previously observed in other cell lines, the retrieved yields after purification differed for recombinant IgMs with higher product titers for IgM617 than IgM012. Indeed, the final purified yield from stable HEK293F culture media of IgM617-HLJ or IgM617HL (5–10 mg/l of culture supernatant) was 25–100 times higher than that of IgM012-HLJ or IgM012-HL (less than 0.1 mg/l of culture supernatant).

### 3.2 Biophysical Characterization of the Oligomeric Forms of Recombinant Immunoglobulins Type-M

The oligomeric distribution and quality of purified IgM samples expressed by HEK293F cell lines were investigated using biochemical, biophysical, and structural methods.

#### 3.2.1 SDS-PAGE

**FIGURE 1:**
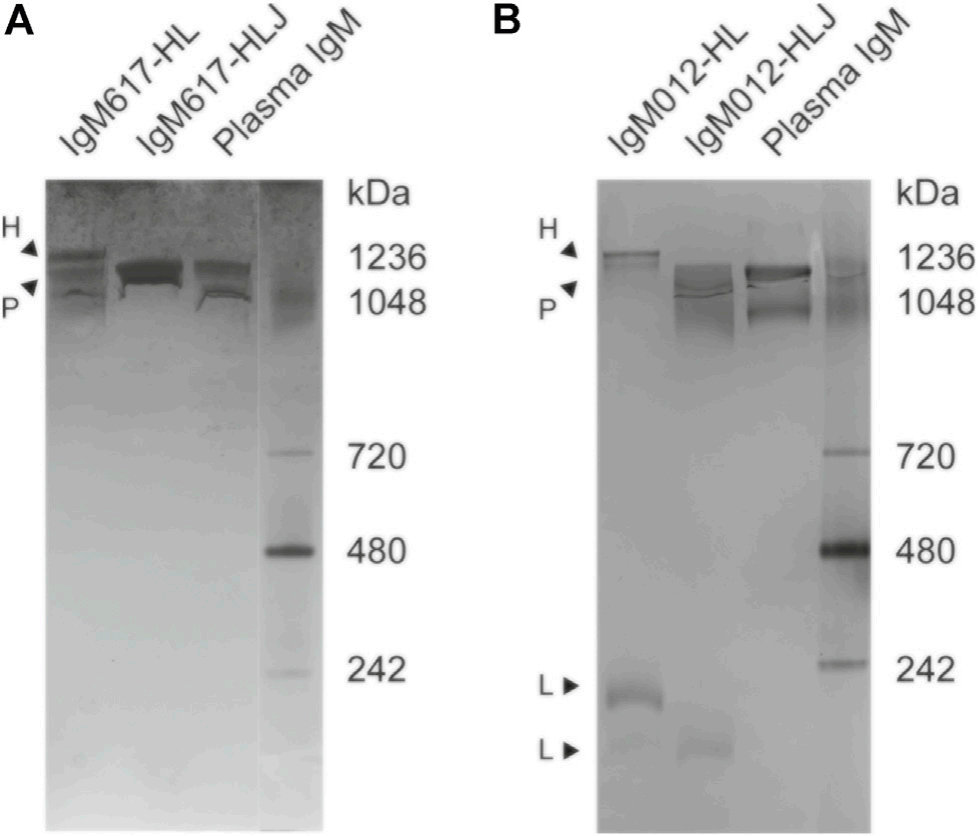
Semi-native polyacrylamide gel electrophoresis (PAGE) analysis of purified IgM samples from HEK293F expression. Native PAGE gels followed by Coomassie Blue staining were run to identify **(A)** IgM617-HLJ and IgM617-HL and **(B)** IgM012-HLJ and IgM012-HL polymer distributions after SEC purification. IgMs purified from plasma (antibodies-online GmbH) are taken as markers along with native markers (Novex NativeMark^TM^ Unstained Protein Standard). Suspected hexamers (H), pentamers (P), and lower assemblies (L) are indicated with arrows.

Semi-native polyacrylamide gel electrophoresis (PAGE) adapted from Vorauer-Uhl et al. (2010) showed that purified IgM617-HLJ migrated as a single and broad band while IgM617-HL migrated in a more heterogeneous manner with two major bands in the highest molecular weight range, suggesting the presence of different high oligomeric states in the absence of J chain (Figure 1A). Interestingly, although IgM012-HLJ and IgM012-HL behaved similarly to IgM617 constructs, additional noticeable bands at molecular weights below 242 kDa were still observed. This suggests the presence of lower assembly states (Figure 1B) as described by Chromikova et al. (2015b) and Hennicke et al. (2020) for the production of IgM617 and IgM012 pentamers in CHO DG44 or HEK239E cell lines.

#### 3.2.2 Size Exclusion Chromatography—Multi-Angle Laser Light Scattering

**FIGURE 2:**
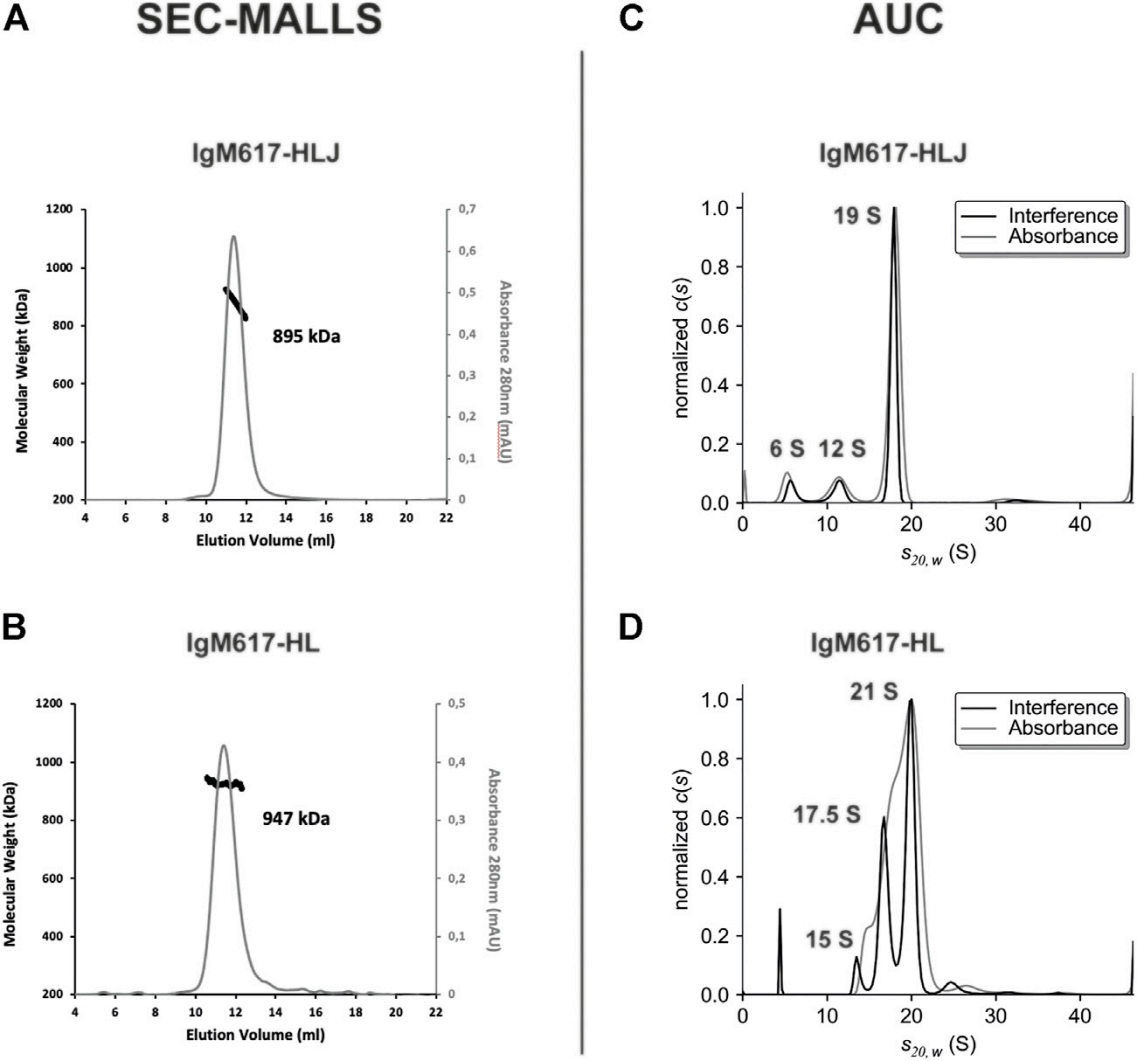
Size exclusion chromatography coupled to multi-angle laser light scattering (SEC-MALLS) and analytical ultracentrifugation (AUC) analysis of purified IgM617 samples from HEK293F expression. The left panels show the elution profiles of the purified **(A)** IgM617-HLJ and **(B)** IgM617-HL monitored by the excess refractive index (right ordinate axis) and the molecular weight as bold line (left ordinate axis) derived from MALLS, refractometry, and UV-Vis measurements. The estimated average molecular weights are indicated on the graphs. The right panels show the sedimentation distributions of **(C)** purified IgM617-HLJ and **(D)** IgM617-HL. Calculated and corrected sedimentation coefficients S_*20,W*_ are obtained as described in Materials and Methods and are indicated on the graphs.

To further characterize IgM617-HLJ and IgM617-HL homogeneity, multi-angle laser light scattering coupled to size exclusion chromatography (SEC-MALLS) was performed. The analyses presented a homogeneous sharp peak of about 895 kDa for IgM617-HLJ, which falls in the range of theoretical molecular weight of the glycosylated pentamers (891 kDa of amino acids +10–15% of glycosylation) (Table 1 and Figure 2A). Surprisingly, a single mass of 947 kDa was also observed for IgM617-HL (Table 1 and Figure 2B), whereas SDS-PAGE analysis showed a mixture of pentamers and hexamers (see above). The observed mass likely corresponds to average molecular weights of the different glycosylated oligomers (hexamers: 1,050 kDa +10–15%; pentamers without J chain: 875 kDa +10–15%). They might have co-eluted as a single peak from SEC, the resolution of this method being insufficient to separate hexamers and pentamers.

**TABLE 1:** Summary of theoretical and experimental molecular weights and of sedimentation coefficients. The theoretical peptide molecular weights are calculated based on the amino-acid primary sequences of the subcloned IgM chains using Protparam (Gasteiger et al., 2005). Experimental molecular weights were determined by SEC-MALLS and MP and sedimentation coefficients by AUC.

IgM	Oligomers	Theoretical (kDa)	SEC-MALLS (kDa)	AUC (S)	Mass photometry (kDa)
**IgM617-HLJ**	Pentamers	891	895	19.0 S	998 ± 6
	Tetramers	700	-	12.0 S	-
	Protomers	175	-	6.0 S	-
**IgM617-HL**	Hexamers	1,050	947	21.0 S	1,250 ± 54
	Pentamers	875		17.5 S	1,050 ± 43
	Tetramers	700	-	15.0 S	841 ± 37
	Protomers	175	-	5.0 S	162 ± 12
**IgM012-HLJ**	Pentamers	878	n.d.	n.d.	944 ± 32
	Tetramers	690	n.d.	n.d.	-
	Protomers	172	n.d.	n.d.	162 ± 14
**IgM012-HL**	Hexamers	1,034	n.d.	n.d.	1,166 ± 21
	Pentamers	862	n.d.	n.d.	1,052 ± 34
	Tetramers	690	n.d.	n.d.	-
	Protomers	172	n.d.	n.d.	150 ± 21

(n.d.: not determined).

#### 3.2.3 Analytical Ultracentrifugation

To further explore sample heterogeneities, sedimentation velocity Analytical ultra-centrifugation (sv-AUC) experiments were performed. Migration in the velocity field of IgM617-HLJ samples showed one main homogeneous peak at a sedimentation coefficient of about 19 S (more than 75%) and minor peaks at 12 S (12%) and 6 S (9%). It reveals the presence of a majority of pentamers but also of lower molecular weight oligomers (Table 1 and Figure 2C). Migration of IgM617-HL samples appeared even more heterogeneous with two main peaks at about 21 S (between 60 and 70% depending on lots) and about 17.5 S (25–30%), which may correspond to hexamers and pentamers, but also with two minor peaks at 15 S (5%) and below 5 S (3–10%) accountable for lower molecular weight oligomers (Table 1 and Figure 2D). Our AUC data on recombinant IgMs are in agreement with the sedimentation coefficients originally measured by Eskeland and Christensen (1975) on purified IgM from patient sera with or without J chain.

#### 3.2.4 Mass Photometry

**FIGURE 3:**
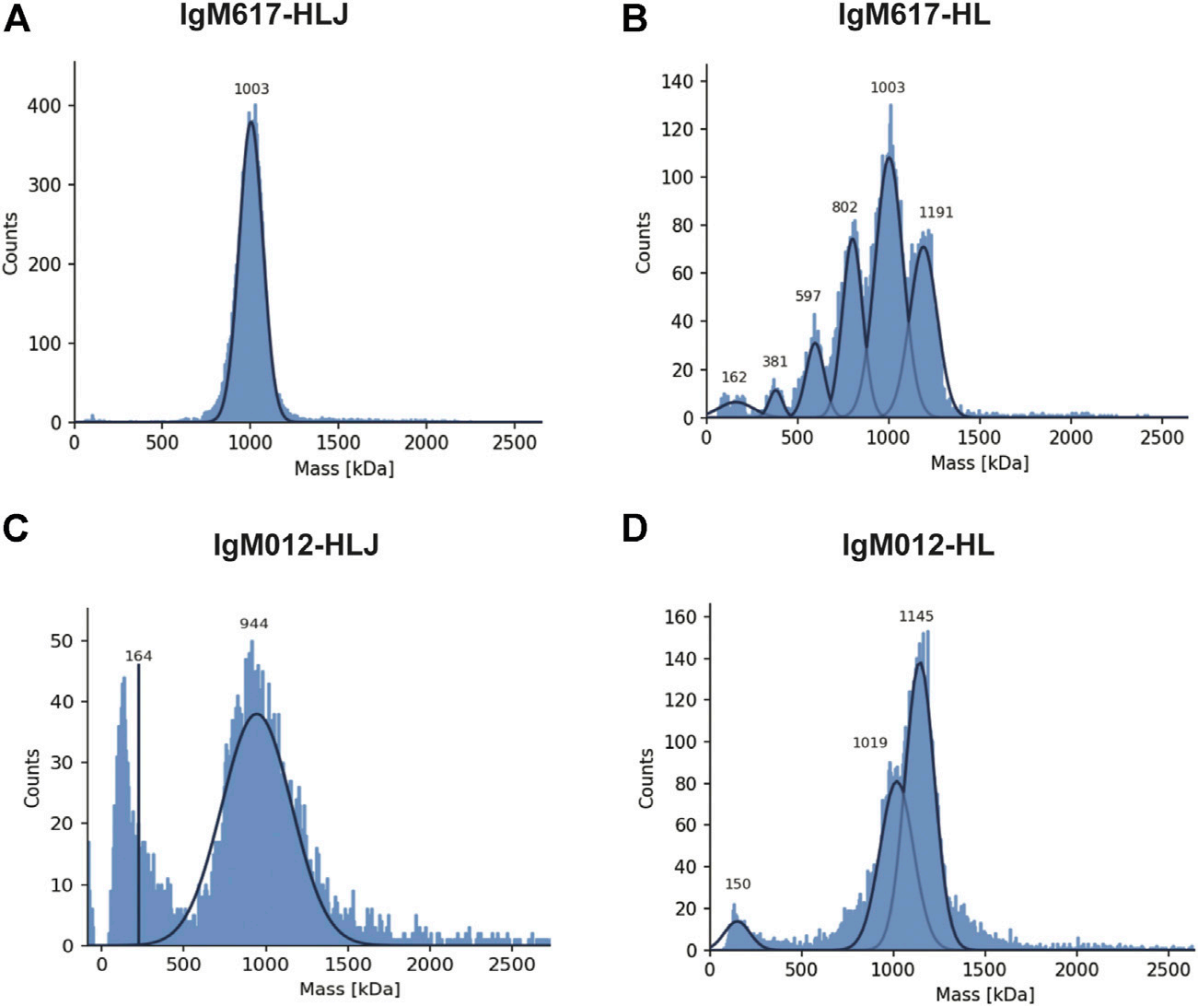
Mass photometry (MP) analysis of recombinant IgMs produced in HEK293F. Histograms show the population distributions of purified IgMs. Shown analyses are representative of replicate experiments: **(A)** IgM617-HLJ, **(B)** IgM617-HL, **(C)** IgM012-HLJ, and **(D)** IgM012-HL. Estimated molecular weights for the shown experiments are indicated on each graph.

To assess more precisely IgM oligomeric states and experimental molecular masses, samples were further analyzed using mass photometry (MP), an emerging technique enabling accurate native mass measurements of single molecules in solution (Sonn-Segev et al., 2020). A single population with an average mass of 998 ± 6 kDa (mean ± SD over replicates) could be observed for IgM617-HLJ (Table 1 and Figure 3A). The experimental mass matched the mass of fully glycosylated pentamers (891 kDa +10–15% glycosylation). By contrast, IgM617-HL data showed three main populations at 1,250 ± 54 kDa, 1,050 ± 43 kDa, and 841 ± 37 kDa (Table 1 and Figure 3B) that might correspond to hexamers (1,050 kDa +10–15% glycosylation), pentamers, (875 kDa +10–15%) and tetramers (700 kDa +10–15%), respectively. Minor populations could also be observed and might correspond to lower oligomeric protomer states. This is in agreement with our sv-AUC observation of the presence of different IgM617-HL oligomers (see above). However, the oligomeric distributions appeared different and might be explained not only by the usage of different batches of IgM617-HL for the measurements but also by the methods used. IgM012-HLJ appeared with a quite homogeneous experimental mass of 944 ± 32 kDa, corresponding to pentamers (878 kDa +10–15%), although a broad mass distribution was still observed and an additional population at low molecular weight was present and might correspond to the lower assembly states (Table 1 and Figure 3C). Finally, IgM012-HL behaved similarly to IgM617-HL with two main populations at 1,166 ± 21 kDa and 1,052 ± 34 kDa corresponding to hexamers (1,035 kDa +10–15%) and pentamers (862 kDa +10–15%), but low assembly states could also be observed (Table 1 and Figure 3D).

#### 3.2.5 Transmission Electron Microscopy

**FIGURE 4:**
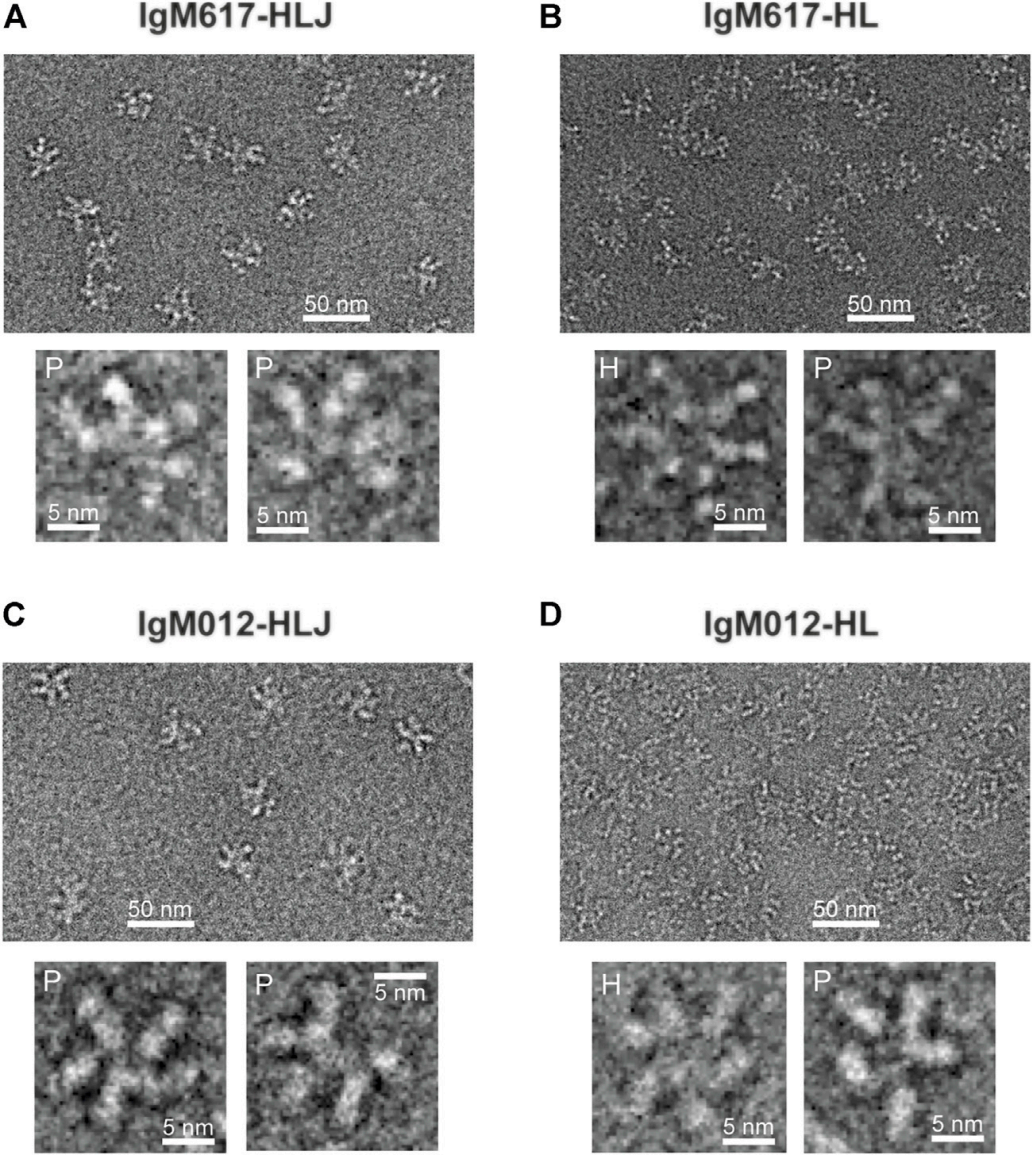
Non-processed images of recombinant IgMs produced in HEK293F by negative stain transmission electron microscopy. Representative fields of particles with a 50 nm scale bar are shown on top of each panel with magnified views of some individual molecules shown on the lower part of each panel: **(A)** IgM617-HLJ, **(B)** IgM617-HL, **(C)** IgM012-HLJ, and **(D)** IgM012-HL. Hexamers are denoted with H and pentamers with P.

The structural integrity and oligomeric states of IgMs produced by HEK293F were also examined and confirmed with negative staining Transmitted electron microscopy (TEM). IgM617-HLJ (Figure 4A) and IgM012-HLJ (Figure 4C) produced in HEK293F exhibited very similar structural characteristics as previously observed for pentamers produced in HEK293E and CHO DG44 (Hennicke et al., 2020). They possessed a central circular core with projecting flexible Fab units in a star-shaped configuration, as well-known for IgMs isolated from human serum. As expected from biophysical data described previously, both IgM617-HL (Figure 4B) and IgM012-HL (Figure 4D) presented two types of distinct particles. Some had a symmetric shape and six arms, confirming the hexameric structural features of HL samples in addition to the pentamer ones, also confirmed by asymmetrical particles to which five arms could be assigned. It should be noted that IgM molecules with lower oligomeric states observed with mass photometry and AUC could not have been easily identified on TEM micrographs.

### 3.3 C1q-Dependent Complement Activation by Recombinant Oligomeric Immunoglobulins Type-M Forms Produced by HEK293F Cells

**FIGURE 5:**
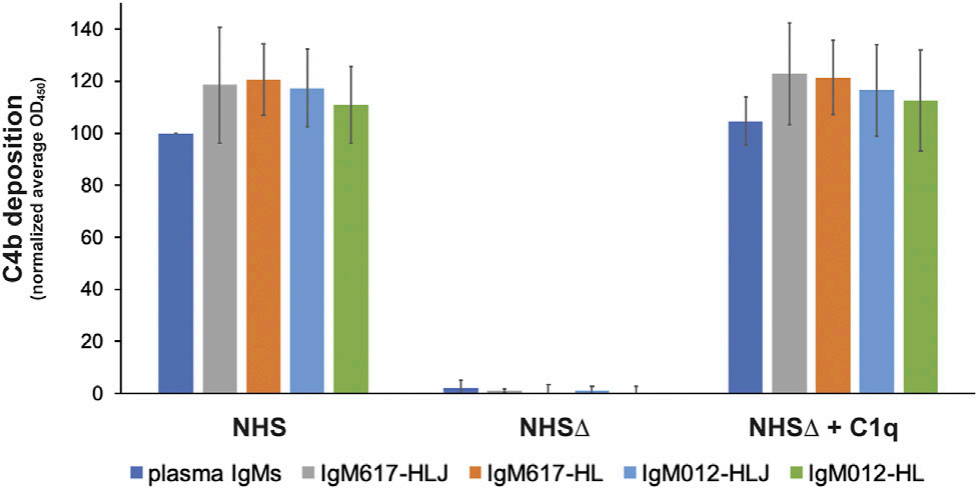
Complement activation by the different purified IgM constructs expressed in HEK293F. Recombinant IgMs and IgMs purified from plasma were coated on microplate wells and incubated with either normal human serum (NHS), C1q-depleted NHS (NHSΔ), or reconstituted NHS (NHSΔ + C1q). C1 activity was monitored *via* C4b deposition. Reported values are average of replicated (2–4) normalized experiments (100% = IgM plasma/NHS). Errors are obtained as standard deviation between independent replicates.

The capacity of the different recombinant IgM preparations to activate CP was analyzed using our in-house *in vitro* enzyme-linked immunosorbent assay (ELISA) based on the detection of C4b fragment deposition after cleavage of C4 by the C1 complex bound to coated IgM molecules (Bally et al., 2019; Hennicke et al., 2020). Although these assays do not confirm antigen-specific CP activation, they allow to demonstrate the coated IgM effector function. Assays with C1q-depleted normal human serum and C1q-reconstituted serum were used as controls for the C1q/IgM interaction dependency. Polyclonal IgMs purified from human plasma (pIgMs) were used as positive control and standard. As observed with our previous IgM617 and IgM012 productions (Hennicke et al., 2020), no significant difference was observed in the C4b deposition yields between the different coated IgM samples, whether serum-derived or recombinant. Thus, the polymer distributions may not influence the ability of IgMs to activate the proteolytic complement cascade through C1 when coated onto the ELISA surfaces (Figure 5).

### 3.4 C1q-Binding Kinetics to Immunoglobulins Type-M Measured by BioLayer Interferometry

In order to characterize the binding kinetics of IgMs to C1q, we developed new protocols using the label-free optical method based on the reflectometric inference spectroscopy (RIfS) (Hänel and Gauglitz, 2002; Gauglitz, 2020) and its setup, known as biolayer interferometry (BLI) (Abdiche et al., 2008; Sultana and Lee, 2015) on an OctetRED96e instrument. In this study, plasma-purified C1q and plasma-derived IgM preparations or recombinant IgM constructs produced in HEK293F were used, in addition to IgMs produced in CHO DG44 and HEK293E (Hennicke et al., 2020). Real-time detection of binding events at the surface of biosensors enables two strategies, using either C1q captured to the biosensor surfaces as ligands and IgMs as analytes or IgMs captured to the biosensor surfaces as ligands and C1q as analytes.

The first strategy was evaluated with C1q ligand either amine coupled onto AR2G biosensors or captured by streptavidin (SA) biosensors after biotinylation. However, although high C1q densities could be reached in both cases (3–7 nm spectral shift), no binding responses could be retrieved with plasma IgM samples (Supplementary Figure S2).

**FIGURE 6:**
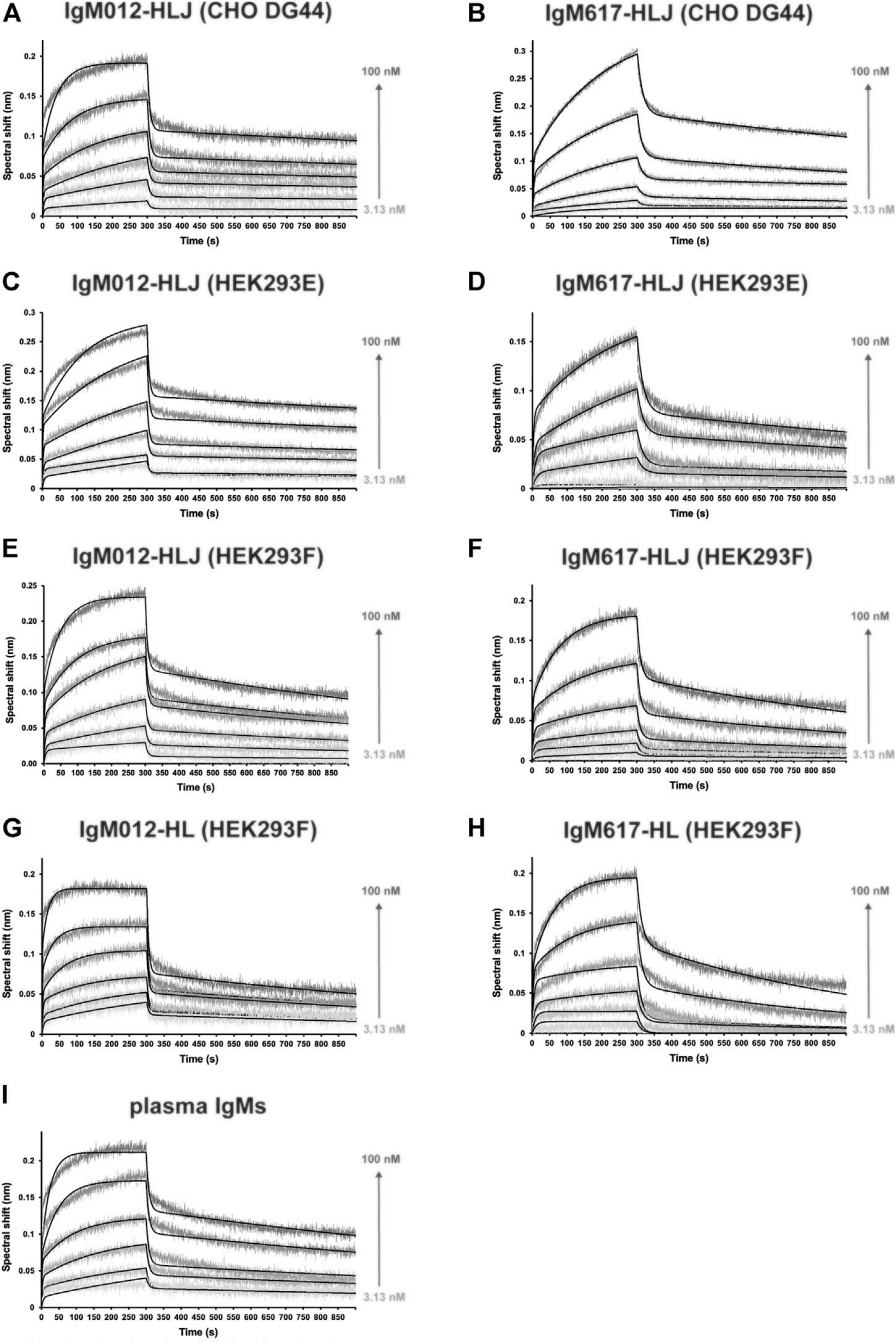
Kinetics analysis of the interaction between C1q from plasma and the different purified and immobilized IgMs expressed in mammalian expression hosts. IgMs were captured on Protein L, and functionalized biosensors were dipped in wells containing C1q at different concentrations (3.13, 6.25, 12.5, 25, 50, and 100 nM). The binding signals (gray-scaled sensorgrams) were obtained by subtracting the signals from empty protein L biosensor and from zero-concentration samples. Fitted curves are depicted as black lines and were obtained by global fitting using a 2:1 heterogeneous ligand model. Shown kinetics analyses are representative of each binding experiment: **(A)** IgM012-HLJ expressed by CHO DG44, **(B)** IgM617-HLJ by CHO DG44, **(C)** IgM012-HLJ by HEK293E, **(D)** IgM617-HLJ by HEK293E, **(E)** IgM012-HLJ by HEK293F, **(F)** IgM617-HLJ by HEK293F, **(G)** IgM012-HL by HEK293F, **(H)** IgM617-HL by HEK293F, and **(I)** IgMs purified from plasma.

In the second strategy, we tested several different commercial biosensors (AR2G, SA, APS, Protein A, and Protein L) and laboratory-made IgM-specific biosensors (mouse or goat anti-μ chain antibodies or CaptureSelect anti-IgM nanobody coated on SA biosensors). All biosensors could be functionalized with plasma-derived polyclonal IgMs at different high levels depending on the capture chemistry (between 1 and 7 nm spectral shift) (Supplementary Figure S3 left panels, Supplementary Figure S3A). Because Protein A is specific for Fcγ regions of IgG but also for VH_3_ subfamilies of Ig, both monoclonal recombinant IgM617 and IgM012 were tested, but no capture signal could be recovered for this type of biosensors, although IgM617 belongs to the VH_3_ subfamily_._ Unspecific binding of C1q was also evaluated for all biosensor types: without any captured IgM, most of them showed either weak or no unspecific signals from C1q, contrary to previous reports (Zhou et al., 2018) (Supplementary Figure S3 right panels). Finally, either no or too weak C1q binding signals could be retrieved from most of the IgM-functionalized biosensors, making them inapplicable for kinetics and affinity determination (AR2G, SA, Protein A, APS, and anti-μ chain antibodies) (Supplementary Figure S3 right panels). Only protein L biosensors showed both limited unspecific C1q binding signals (Supplementary Figure S4B), and measurable kinetics signals on the IgM-functionalized biosensors. Therefore, pIgMs, IgM617, and IgM012 constructs were stably absorbed on Protein L biosensors until saturation was reached (4–7 nm shift) (Supplementary Figure S4B) and kinetics analyses were then performed by dipping the IgM-functionalized biosensors in C1q concentration series ranging from 3.13 to 100 nM with 2-fold dilution (Figure 6).

C1q bound to all IgMs in a biphasic manner, with two distinguishable fast and slow binding rates, and the 1:1-Langmuir model could not be applied to determine interaction kinetics parameters. The only fitting model available to account for the complicated kinetics components was the 2:1-heterogeneous-ligand model (Figure 6). Plasma-derived polyclonal and all recombinant monoclonal IgMs showed affinities in the nanomolar scale, ranging from about 1 to 8 nM for K_D1_ and from 4 to 26 nM for K_D2_, depending on the sample (Table 1). pIgMs (K_D1_ = 2.3 nM and K_D2_ = 6.5 nM) and IgM012s (K_D1_ = 1–3 nM and K_D2_ = 4–10 nM) affinities for C1q appear higher than IgM617s (K_D1_ = 6–8 nM and K_D2_ = 7–26 nM). However, those differences may not be significant. It should be noted that for all IgM samples, the distinct slow and fast kinetics led to affinities of the same nM magnitude. No significant differences in kinetics, which could be related to the host cell lines and the differential glycosylation patterns induced by the expression system (Hennicke et al., 2020), could be observed either for IgM617 or IgM012 models. Finally, although small differences could be noted in kinetics rate values when comparing HL and HLJ constructs, it is difficult to attribute them solely to the presence of hexameric forms, since such variations are also observed either between HLJ constructs or between different preparations of the very same recombinant IgM construct (Table 1).

## 4 Discussion

In the presented study, we recombinantly produced and purified two IgM models, IgM617 and IgM012 (Hennicke et al., 2017, 2019, 2020), and fully characterized their biochemical and functional quality attributes using combined biophysics techniques: AUC, SEC-MALLS, MP, and TEM to determine their oligomeric states and polymer distributions and *in vitro* complement activation based on ELISA to evaluate their functional capacities. Moreover, we used the label-free and real-time detection BLI technique and adapted protocol to quantify the kinetics of the interaction between IgMs and C1q, the recognition and activating protein of the classical complement pathway.

One challenge for studying IgM and developing potential therapeutic products is the manufacturing and characterization of recombinant molecules with high Ig productivity, high homogeneous oligomerization status and purity, and similar biological biomimetic function. In the past, several methods have been tested. In particular, IgM617 and IgM012 have been taken as IgM models to develop proofs-of-concept of recombinant production (Vorauer-Uhl et al., 2010; Mader et al., 2013; Chromikova et al., 2015b; Hennicke et al., 2017, 2019, 2020). In the present study, we complemented the panel of biophysical analysis methods by using SEC-MALLS (Figure 2), sv-AUC (Figure 2), and the new MP (Figure 3) to determine the oligomeric distribution of recombinant IgM samples produced in HEK293F cell line. We can thus confirm that IgM617-HLJ model can be produced as very homogeneous pentamers when H, L, and J chain genes are transfected in HEK293F for stable expression, while IgM012-HLJ retains heterogeneities with significant amounts of lower assemblies. Nevertheless, both IgM pentamers produced in HEK293F retain their structural integrity of five-branches-star-shaped particles (Figure 4) as shown with negative staining TEM images and functional qualities as demonstrated with complement activation assays (Figure 5). One explanation for the low expression of IgM012 in recombinant expression compared to IgM617 and in general to other IgM constructs studied so far may come from the structural arrangement of its Fab. Indeed, the crystal structure of HIV-1 monoclonal antibody 2G12 Fab has revealed an unusual VH-VL domain arrangement for Fabs in which two of its Fabs assemble as a dimer with interlocked VH domain swapping, instead of the usual VH-VL monomeric assembly (Calarese et al., 2003). The VH domain exchange has been demonstrated to be mandatory for this IgG to target and recognize the specific high-mannose cluster of the glycan shield of HIV-1 (Doores et al., 2010). One can easily hypothesize that IgM012 may possess such a similar domain swapping of its Fabs and that this may constrain IgM assembly more than the regular assembly in which Fabs behave independently. These structural constraints may not be favorable and may prevent proper expression of the recombinant IgM012 constructs by the different cell lines used so far.

With this study, we show that hexamer expression of IgM617-HL, and to a lesser extent of IgM012-HL, is made possible using HEK293F stably transfected with only the H and L constructs. It was well established that the J chain expression influences the formation of the pentameric and hexameric IgM forms either *in vivo* or *in vitro*. Randall et al. (1992) first demonstrated the regulation of the pentamer/hexamer ratio in B cells by J chain gene expression with the secretion of a majority of hexamers and a minority of pentamers by cells lacking the J-chain gene. However, recombinant expression of only hexamers by J-chain-deficient cells remains challenging. Although hexamers were found to be expressed by recombinant systems, a majority of pentamers was found to be secreted by mouse hybridoma cell lines (Wiersma et al., 1998; Collins et al., 2002) or CHO-K1 (Gilmour et al., 2008). Interestingly, only Azuma et al. (2007) have succeeded to obtain a majority of hexamers by using CHO DG44 that express 20 times more hexamers than CHO co-transfected with the J-chain gene. Here, we confirm that recombinantly producing homogeneous hexameric forms of IgM remains difficult. Although IgM617 and IgM012 models can be expressed and secreted as hexamers by stable HEK293F cell lines, as shown by our different biochemical, biophysical, and structural analyses, our data also show that both sample types are not homogeneous. They present a large population of pentamers likely lacking the J chain, but also with traces of lower molecular-weighted tetramers and dimers in the purified IgM samples. Furthermore, the ratio of the distinct IgM oligomeric states appears to vary not only between IgM models but also between different production runs of the same IgM model (data not shown). In addition, data from our surface-based C1q-dependent complement activation assays do not demonstrate higher activities of hexameric-enriched IgM samples (Figure 5). This is in contrast to previous hemolytic assays, which have demonstrated the higher efficacy of hexameric IgM in inducing complement-dependent cytolysis compared to pentameric IgMs (Randall et al., 1990; Collins et al., 2002). As previously mentioned, the ELISA setup and the end-point measurement of C4 deposition may not be sensitive and resolutive enough to characterize differences in IgM abilities to activate the first step of complement activation (Hennicke et al., 2020).

For the first time, we present the new usage of BLI to characterize the binding kinetics and affinities of C1q to IgM antibody isotypes. Optical and surface-based methods such as SPR and BLI are popular methods for characterizing the binding properties of all antibody classes to antigens, but much less so to Ig effectors such as the complement molecule, C1q. Patel et al. (2015) and Jovic and Cymer (2019) have successfully characterized the C1q binding responses to the different IgG subtypes using SPR methods. Zhou et al. (2018) did the same using the BLI technology. Only one study has been published by Bally et al. (2019), who measured the binding kinetics between IgM fractions purified from human plasma and native C1q, as well as recombinant C1q, and a few critical mutants, using SPR.

Several experimental setups were tested in our study to define the conditions allowing reliable binding measurements between either IgM purified from human plasma or different recombinant IgM constructs and native C1q purified from human plasma. Surprisingly, and in contrast to the SPR data obtained so far when C1q was captured at the biosensor surface (Bally et al., 2019), no interaction with IgMs was detectable with the BLI method (Supplementary Figure S2). However, we were able to observe binding of the catalytic tetramer C1r_2_Cs_2_ to immobilized C1q with expected affinities in the nanomolar range (Supplementary Figure S5). These results indicate that immobilized C1q can retain some binding activities towards its partners, but that the immobilization strategy onto the BLI biosensor, either directly or by selective biotinylation of primary amines of C1q, can greatly affect its binding capabilities.

During optimization of the BLI protocols, capturing IgMs onto Protein L biosensor and using C1q as analyte proved to be the most efficient strategy since only weak or acceptable C1q unspecific binding could be detected at the used concentration range (Supplementary Figure S4) and specific concentration-dependent binding signals could be measured (Figure 6). Our method is similar to those already employed to characterize the complexes between C1q and recombinant IgGs, for which Protein L-coated sensors have been used to capture immunoglobulins for SPR or BLI, although streptavidin and biotin fusion also proved to be suitable (Zhou et al., 2018). Unfortunately, protein L was the only successful immobilization method for functionalizing BLI biosensors with IgMs. Indeed, the binding of protein L to Igs is specifically restricted to some kappa light chains, which limits the application of the method described here to IgMs containing this subtype of the L chain. Surprisingly, the binding of C1q could be measured in those conditions and without any specific antigen bound to IgMs. Similarly, complement activation could be performed by binding IgM to microtiter plates without antigen with solid-phase methods such as ELISA techniques to prove the effector function capacity of IgMs (Zwirner et al., 1998; Bally et al., 2019; Hennicke et al., 2020), whereas antigen is required in solution methods such as erythrocyte or liposome lysis (for assays review in Harboe et al., 2011). Indeed, C1q binding to IgM and subsequent activation of the complement cascade are thought to occur physiologically only with IgM binding to a specific antigen. This latter may induce large structural changes in IgM quaternary structure and exposure of the hidden binding sites for C1q globular heads (Sharp et al., 2019). One possible explanation would be that immobilizing IgMs onto an *in vitro* surface might provoke the necessary structural feature exposures. In particular, for BLI experiments for which only IgM capture *via* Protein L allowed C1q binding, the explanation might come from the binding properties of Protein L to Ig. As shown by the 3D structure of the complex between Protein L and Fab, one Protein L domain bridges two Fab domains (Graille et al., 2001). Although this property may not strictly mimic antigen binding, it might be enough to induce an IgM conformation allowing complex formation with C1q.

As expected, the measured affinities of C1q for IgMs with BLI fall within the nM range (Table 2). No significant differences in affinities and kinetics rates could be observed between polyclonal IgM purified from plasma and the different recombinant monoclonal IgMs, regardless of the host cell lines, oligomeric distributions, or post-translational glycosylation pattern (Hennicke et al., 2020). Interestingly, C1q affinities for IgMs are in the same nM range as for IgG1 measured in a similar BLI configuration, although kinetics behavior differs slightly (Zhou et al., 2018). Together with the complement assays, which also showed no differences between IgM samples, our BLI data emphasize that recombinant IgM preparations retain similar abilities as physiological IgMs to bind and activate the CP *in vitro*. Our data are consistent with SPR data obtained by Bally et al. (2019) since the affinities of IgMs for C1q measured by SPR and BLI are very similar. However, the kinetics behavior appeared to be different since a 2:1 heterogeneous model was applied to fit our BLI binding data (Figure 6), while a 1:1 Langmuir model was sufficient to interpret SPR data. One can argue that the biphasic kinetics behavior may be raised by the heterogenous quality of native or of our recombinant IgM samples. However, no difference was observed between the most homogeneous constructs (IgM617-HLJ) and the other constructs. Thus, the differences between SPR and BLI data likely originate from either the used strategies or methods. Indeed, the employed sensor functionalization strategies were different with different capture chemistries and captured molecules (amine coupled C1q in case of SPR and Protein L/IgMs in case of BLI) and different molecules are used as analytes (IgM in case of SPR and C1q in case of BLI). The question of the used method will remain unanswered since no binding could be detected with SPR, capturing IgMs on Protein L sensors and using C1q as analytes (data not shown). The biphasic kinetics behavior of the IgM/C1q binding measured with BLI might also come from their intrinsic molecular characteristics, such as their highly oligomeric states and their flexibility. Indeed, it is expected that the single globular region of C1q may have a lower affinity for Igs than full C1q, as well as IgM protomer for C1q than full IgM. Thus, the affinities for the C1q/IgM complex formation rely on the specific multivalency that enhances the binding with an avidity effect. Furthermore, certain lability of the C1q/Ig complex formation can also influence kinetics binding. Not all gC1q bind the IgG hexamers at the same time (Ugurlar et al., 2018), although the same behavior has not been described for IgMs (Sharp et al., 2019). Finally, due to the very high flexibility of IgM and C1q molecules, structural arrangements and stabilization are expected to have an effect on the dynamics of binding events. Taken together, these molecular features would induce very complicated and heterogeneous binding kinetics for which any mathematical fitting model would not be sufficiently formulated and easy to apply to consider all possible behaviors.

**TABLE 2:** Kinetic and affinity constants of C1q binding to different purified recombinant IgMs expressed in mammalian expression systems. Values are obtained after global fitting of the binding signals (Figure 5) and averaging from replicates (2–4). Affinity constants (K_D1_ and K_D2_) are obtained from the ratio between kinetics parameters (k_d1_/k_a1_ and k_d2_/k_a2_). Standard errors are obtained as standard deviation between replicates.

IgM	Source	Construct	k_a1_	k_d1_	K_D1_	k_a2_	k_d2_	K_D2_
			10^5^/Ms	10^−4^/s	10^−9^ M	10^7^/Ms	10^−1^/s	10^−9^ M
**Total IgM**	Plasma		3.04 ± 0.47	7.02 ± 1.63	**2.31 ± 0.09**	2.00 ± 0.90	1.30 ± 0.60	**6.52 ± 1.34**
**IgM617**	CHO DG44	HLJ	0.78 ± 0.17	6.20 ± 1.02	**8.00 ± 0.31**	0.57 ± 0.44	0.74 ± 0.08	**12.30 ± 3.87**
	HEK293E	HLJ	0.92 ± 0.16	6.26 ± 1.77	**6.82 ± 0.11**	0.86 ± 0.49	0.59 ± 0.33	**6.90 ± 1.13**
	HEK293F	HLJ	1.59 ± 0.76	11.30 ± 3.04	**7.10 ± 1.06**	0.44 ± 0.05	1.15 ± 0.53	**26.4 ± 2.89**
	HEK293F	HL	1.66 ± 0.50	9.25 ± 4.84	**5.59 ± 1.02**	0.96 ± 0.38	0.96 ± 0.47	**9.97 ± 2.00**
**IgM012**	CHO DG44	HLJ	2.22 ± 0.08	2.57 ± 0.37	**1.16 ± 0.01**	2.04 ± 0.31	1.34 ± 0.13	**6.54 ± 0.11**
	HEK293E	HLJ	1.64 ± 0.35	3.57 ± 0.33	**2.18 ± 0.06**	1.84 ± 0.40	1.57 ± 0.15	**8.51 ± 0.24**
	HEK293F	HLJ	2.22 ± 0.33	6.18 ± 0.08	**2.79 ± 0.03**	1.41 ± 0.34	1.34 ± 0.28	**9.50 ± 0.48**
	HEK293F	HL	4.65 ± 2.54	5.81 ± 2.61	**1.25 ± 0.31**	1.79 ± 0.51	0.73 ± 0.04	**4.09 ± 0.17**

In conclusion, our work presents relevant results for the development of IgM as biopharmaceuticals. This latter requires new *in vitro* methods to produce biosimilars and to characterize the quality attributes of recombinant samples to meet the regulations of various drug and health authorities around the world. For antibodies, non-clinical studies must be performed to assess first the product qualities in terms of purity and homogeneity. For example, electrophoresis is widely used so far but with the known limitations in detecting low protein amounts and the application of particular protocols necessary to characterize high molecular-weight biomolecules such as IgMs (Vorauer-Uhl et al., 2010). Our results show the relevance of additional biophysical methods to precisely determine oligomeric distributions and the molecular weight of different IgM sample states. In particular, mass photometry appears to be a reliable technique to assess rapidly the mass distribution with minimal sample amounts. The characterization of the similarity in known Fab- and Fc-associated functions such as binding to target antigen(s) and to Fc gamma receptors, FcRn, or C1q is challenging, in particular, in finding methods to demonstrate the binding of Igs to C1q. For now, ELISA has been widely used as a surrogate for complement-dependent cytotoxicity assays in comparability studies for therapeutic Igs. SPR and BLI have proven to be alternatives with advantages like lower complexity in buffer preparations, lower hands-on manipulation, and lower sample consumption. They are also faster, more in-depth, and semi-automated interaction analysis methods with higher precisions than the endpoint measurements from ELISA (Patel et al., 2015; Zhou et al., 2018; Jovic and Cymer, 2019). With our study, we demonstrate the suitability of the BLI-based assays for the measurements of recombinant IgM/C1q interactions, and we believe that the developed protocols can be easily applied to evaluate future IgM potential therapeutic preparations in combination with the conventional biological analysis.

## Data Availability

The original contributions presented in the study are included in the article/Supplementary Material, further inquiries can be directed to the corresponding author.

## Abbreviations

sv-AUC/AUC, sedimentation velocity analytical ultra-centrifugation; BLI, biolayer interferometry; CLR, collagen-like region of C1q; ELISA, enzyme-linked immunosorbent assay; H/H chain, heavy chain; L/L chain, light chain; gC1q, globular head of C1q; Ig/Igs, immunoglobulin/s; IgG/IgGs, immunoglobulin/s G; IgM/IgMs, immunoglobulin/s M; J/J chain, joining chain; EM, electron microscopy; MP, mass photometry; PCR, polymerase chain reaction; RIfS, reflectometric interference spectroscopy; SEC, size exclusion chromatography; SEC-MALLS, multi-angle laser light scattering coupled to size exclusion chromatography; SPR, surface plasmon resonance; TEM, transmission electron microscopy; PAGE, polyacrylamide gel electrophoresis.
