# Supplementary material for: Biophysical Characterization of the Oligomeric States of Recombinant Immunoglobulins Type-M and Their C1q-Binding Kinetics by Biolayer Interferometry
Source: Front Bioeng Biotechnol. 2022 May 24;10:816275. doi: 10.3389/fbioe.2022.816275 (PMC9173649; doi:10.3389/fbioe.2022.816275)
Supplement: Supplementary file 1 [file DataSheet1.PDF]

Supplementary Figure S1

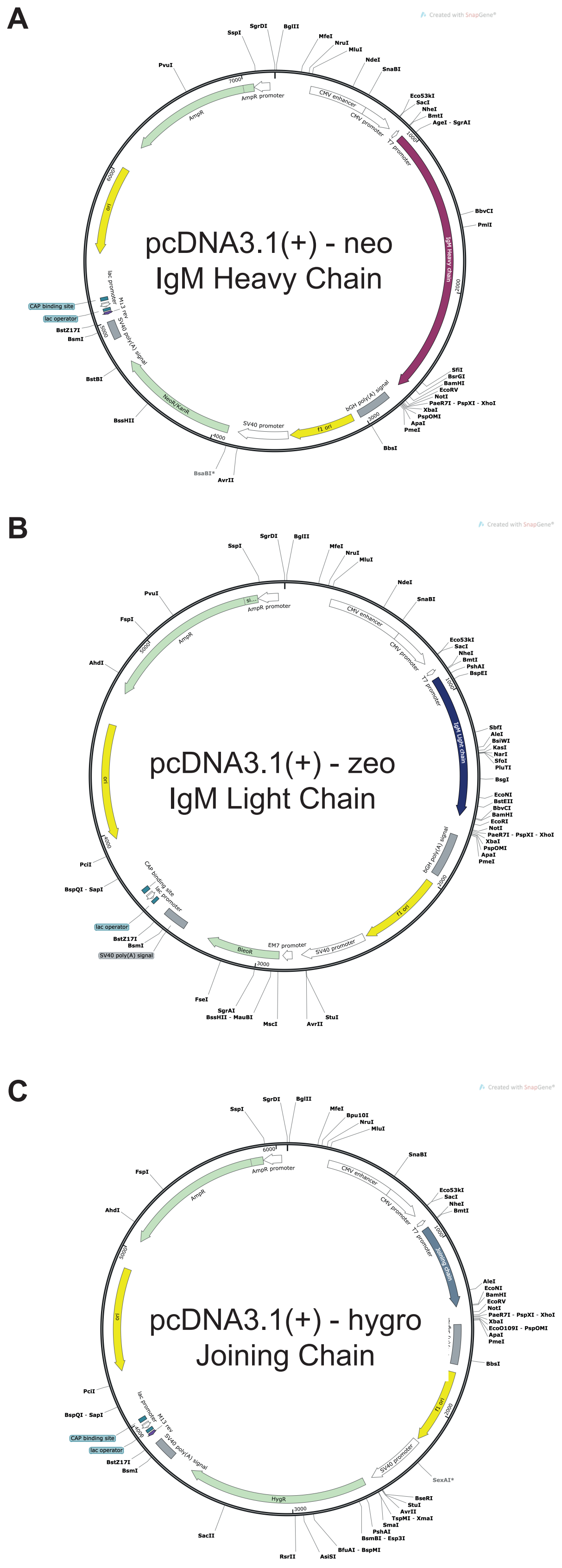

Supplementary Figure S1. Plasmid maps of pcDNA3.1(+) constructs used to obtain stable HEK293F cell lines expressing IgM617-HL, IgM617-HLJ, IgM012-HL and IgM012-HLJ.

Supplementary Figure S2

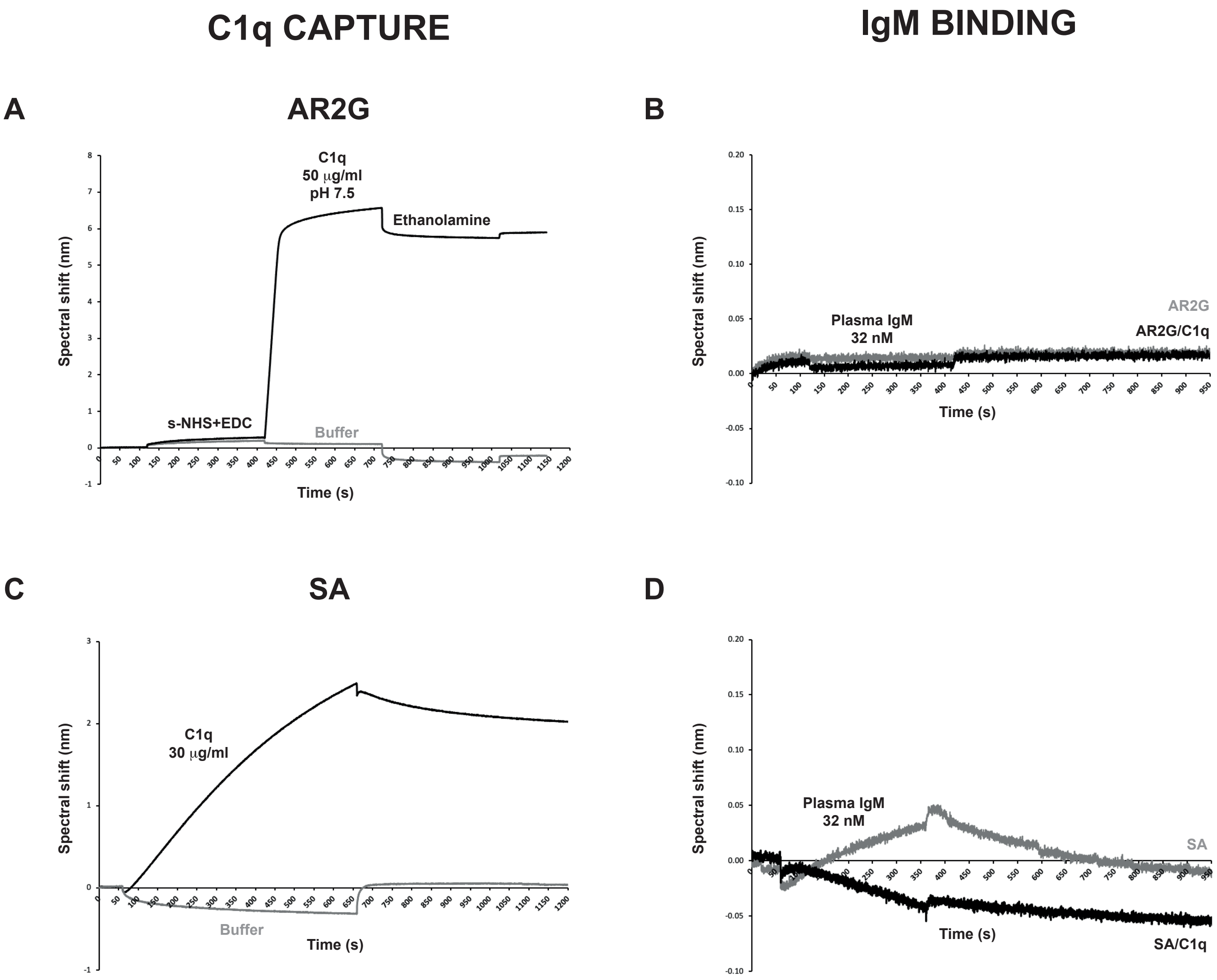

Supplementary Figure S2. Examples of C1q captures on (**A**) AR2G or (**C**) SA biosensors. (**B and D**) and examples of plasma IgM bindings at 32 nM to functionalyzed biosensors (in black) or reference biosensors (in grey).

Supplementary Figure S3

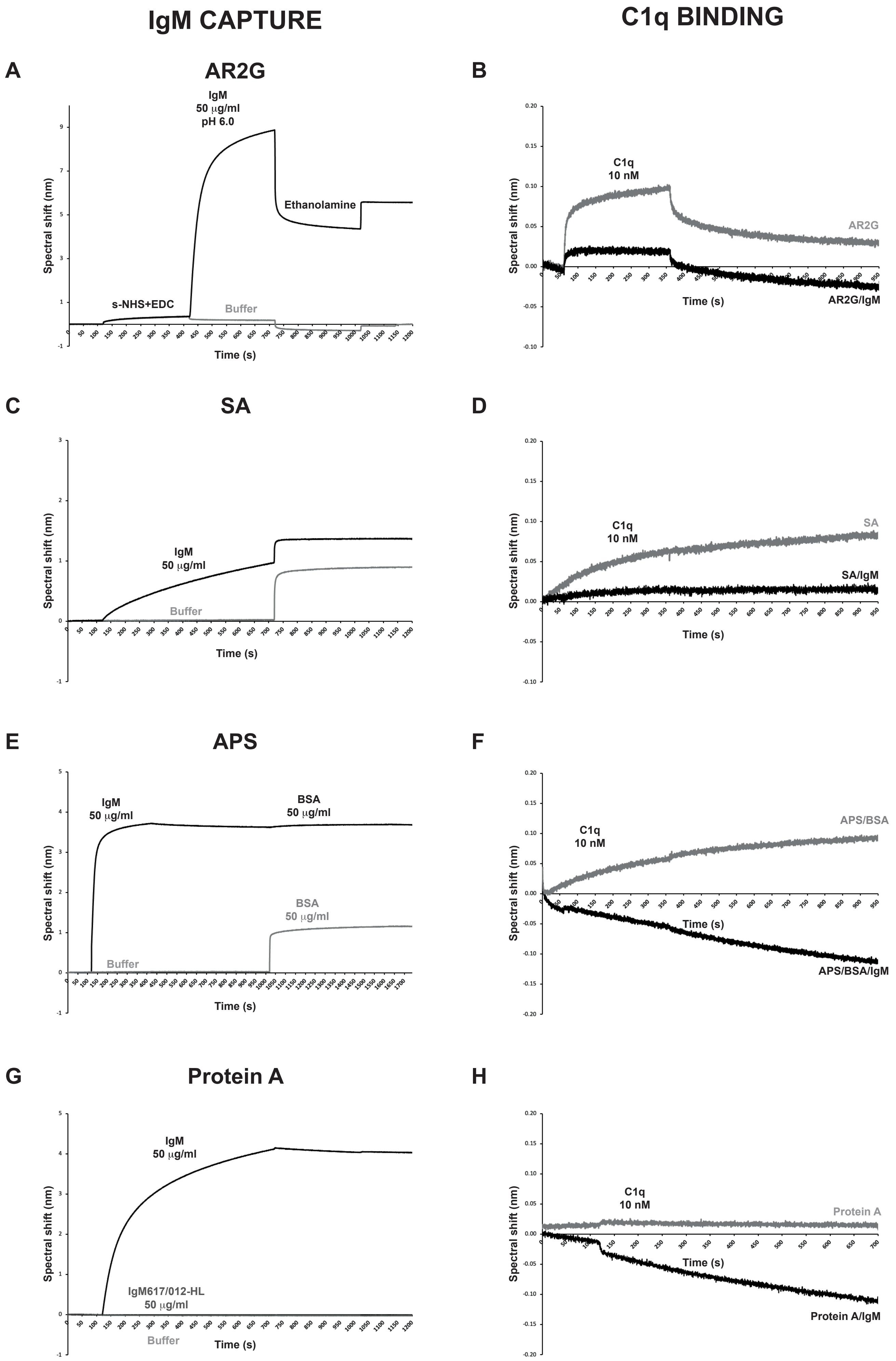

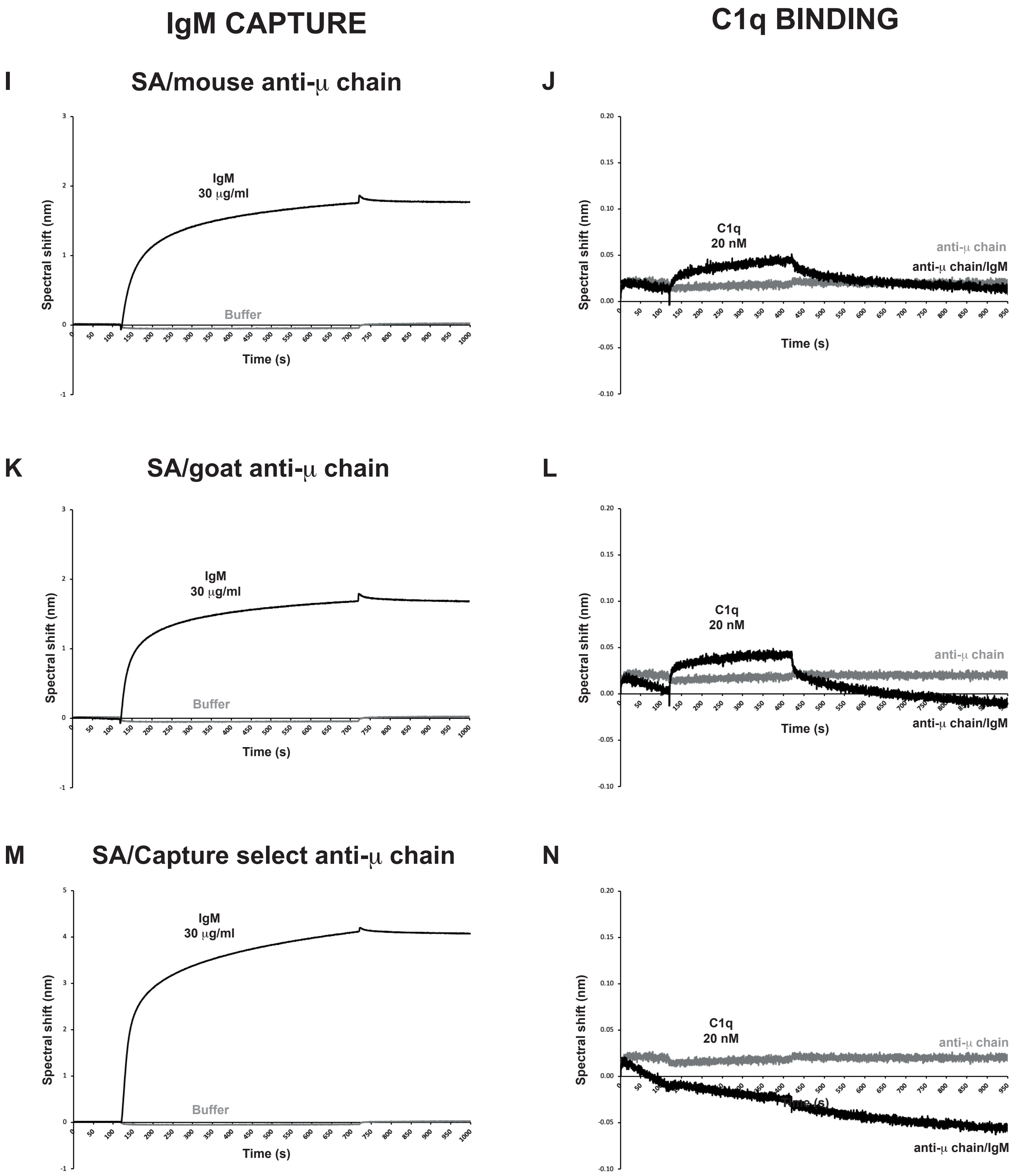

Supplementary Figure S3. Examples of plasma IgM captures on (**A**) AR2G, (**C**) SA, (**E**) APS, (**G**) Protein A, (**I, K, M**) anti-μ chain biosensors and (**B, D, F, H, J, L, N**) of plasma C1q binding at 10 or 20 nM to functionalized (in black) and reference (in grey) biosensors.

Supplementary Figure S4

IgM capture **C1q unspecific binding**

A **B**

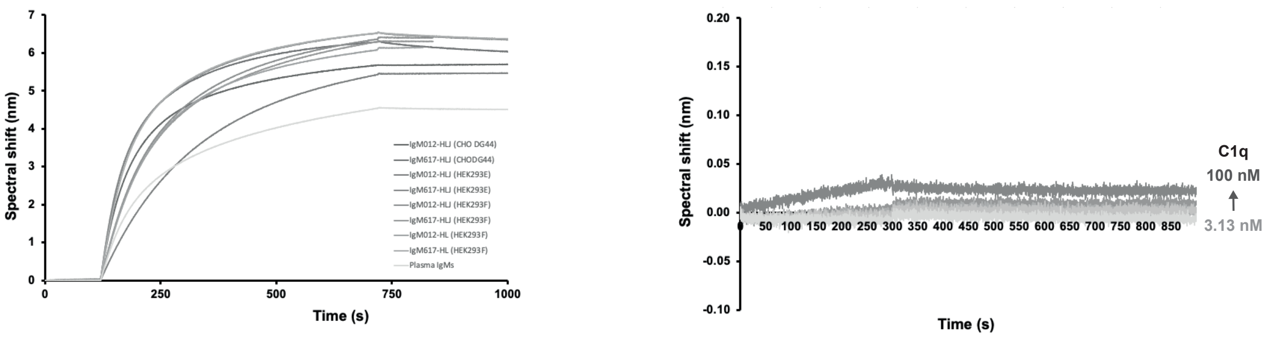

Supplementary Figure S4. (**A**) Capture of IgM samples onto Protein L biosensors (IgM concentration = 30 µg/ml). (**B**) C1q unspecific binding on Protein L biosensor at different concentrations from 3.13 nM to 100 nM.

Supplementary Figure S5

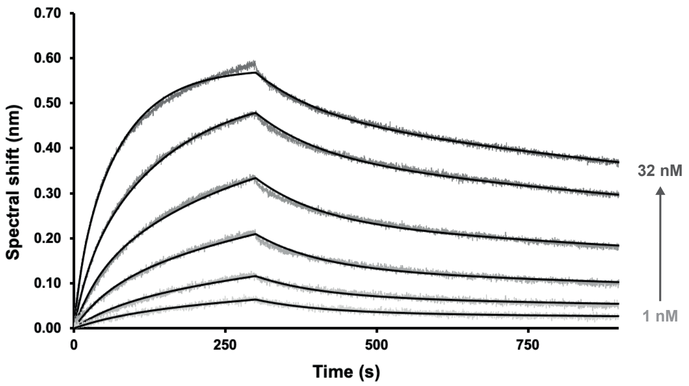

k_{a1} 10^5/Ms	k_{d1} 10^{-4}/s	**K_{D1} 10^{-9} M**	k_{a2} 10^6/Ms	k_{d2} 10^{-3}/s	**K_{D2} 10^{-9} M**
4.06 ± 0.01	4.02 ± 0.03	**10.00 ± 0.07**	2.14 ± 0.02	9.28 ± 0.01	**4.33 ± 0.01**

Supplementary Figure S5. Kinetics analysis of the interaction between C1q from plasma and recombinant $C1r_2C1s_2$. C1q was immobilized on AR2G biosensor (supplementary Figure S1). The tetramer was expressed in mammalian cells (Bally et al., 2019). The functionalized biosensors were dipped in wells containing $C1r_2C1s_2$ at different concentrations (1, 2, 4, 8, 16, 32 nM). The binding signals (grey-scaled sensorgrams) were obtained by subtracting the signals from empty AR2G biosensor and from zero-concentration samples. Fitted curved are depicted as black lines and were obtained by global fitting using a 2:1 heterogenous ligand model. Kinetics parameters and affinities are reported in the table.
